# Complementary Powerful Techniques for Investigating the Interactions of Proteins with Porous TiO_2_ and Its Hybrid Materials: A Tutorial Review

**DOI:** 10.3390/membranes12040415

**Published:** 2022-04-11

**Authors:** Yihui Dong, Weifeng Lin, Aatto Laaksonen, Xiaoyan Ji

**Affiliations:** 1 Department of Molecular Chemistry and Materials Science, Weizmann Institute of Science, Rehovot 76100, Israel; lin.weifeng@weizmann.ac.il; 2 Energy Engineering, Division of Energy Science, Luleå University of Technology, 97187 Luleå, Sweden; aatto.laaksonen@mmk.su.se; 3 Arrhenius Laboratory, Department of Materials and Environmental Chemistry, Stockholm University, 10691 Stockholm, Sweden; 4 Center of Advanced Research in Bionanoconjugates and Biopolymers, ‘‘Petru Poni” Institute of Macromolecular Chemistry, 700469 Iasi, Romania; 5 State Key Laboratory of Materials-Oriented and Chemical Engineering, Nanjing Tech University, Nanjing 211816, China

**Keywords:** porous materials, TiO_2_, high-performance liquid chromatography (HPLC), atomic force microscopy (AFM), surface enhanced Raman scattering (SERS), molecular dynamics (MD) simulations

## Abstract

Understanding the adsorption and interaction between porous materials and protein is of great importance in biomedical and interface sciences. Among the studied porous materials, TiO_2_ and its hybrid materials, featuring distinct, well-defined pore sizes, structural stability and excellent biocompatibility, are widely used. In this review, the use of four powerful, synergetic and complementary techniques to study protein-TiO_2-_based porous materials interactions at different scales is summarized, including high-performance liquid chromatography (HPLC), atomic force microscopy (AFM), surface-enhanced Raman scattering (SERS), and Molecular Dynamics (MD) simulations. We expect that this review could be helpful in optimizing the commonly used techniques to characterize the interfacial behavior of protein on porous TiO_2_ materials in different applications.

## 1. Introduction

Proteins interacting with surfaces of various substrates play a pivotal role in bioengineering and medicine and are of fundamental importance for developing new nanotechnologies and designing nanomaterials for biological applications [1]. Understanding the adsorption and interactions between porous materials and biosystems is critical in environmental and biomedical sciences. One of the most investigated porous materials, TiO_2_, has been extensively used in many industrially relevant applications ranging from environment to life sciences due to its efficient photoactivity, high chemical/thermal stability, and low cost [2]. Although TiO_2_ has been intensively studied in various fields, using TiO_2_ in bio-related applications is much more attractive [3]. This is because TiO_2_ is a favorable biomaterial for manipulating biomolecules due to its excellent biocompatibility [3], controllable structural properties (morphology, crystal form, chemistry), as well as its long-term stability compared to other widely-used materials (i.e., porous silica [4], nanoporous sol-gel arrays [5]). The first studies were reported in the 1990s owing to the use of titanium in implants and the need to understand the interaction of biomolecules with implant surfaces covered with oxidized titanium [6,7]. Soon afterwards, this field developed quite quickly, and the number of publications began to exponentially increase. TiO_2_ interacting with biomolecules establishes a series of nanoparticle/biological interfaces, and their properties and process performance depend on a combination of different physicochemical interactions. Probing and determining these various interfacial interactions allows the development of predictive relationships between structure and properties/performance.

When these porous materials were introduced to the biological field, controlling the interaction between porous materials and biomolecules became a challenging problem for different applications, such as bio-separation, biosensors, drug delivery, and bio-detection [1,8]. It is widely accepted and recognized that the adsorption capacity of proteins provides a general criterion for determining the interaction strength between the protein and the adsorbed surfaces [9,10,11,12]. The widely and directly used approaches to control the interactions is through controlling the adsorption capacity. However, the adsorption capacity alone is not always a good indicator of interaction strength because the adsorption amount does not always correctly reflect effective protein surface attachment. More in-depth understanding and characterizations of the interactions between proteins and porous materials are needed for investigating interfacial interactions.

It is well-known that the performance of proteins separation/purification using chromatography mainly relies on regulating the protein interaction with related surfaces. The retention behavior test by chromatography can qualitatively represent the interaction strength between the protein and the solid surfaces. Thus, high performance liquid chromatography (HPLC) has been used as a reliable technique to study the interaction strength of proteins with the column materials at the macroscale [13]. However, the chromatographic retention time is unable to provide the information on molecular interactions at the nanoscale [14,15], and alternative options are needed to determine the interactions at the nano/microscale.

Atomic Force Microscopy (AFM), which possesses sensitivity at the nanoscale, can achieve molecular resolution of adsorbed molecules and directly reflect the atomic/molecular-level interactions [16]. This technique has undoubtedly made a considerable impact on life sciences in characterizing and manipulating biological interfaces [16,17], and provides both quantitative and dynamic information about protein interaction at the nanoscale [18,19]. Many studies have been conducted on evaluating protein interactions on a biomaterial surface [20,21,22,23,24,25]. The AFM-measured interaction force is related to the effective contact area between the AFM-tip and the related surfaces [26]. Depending on the number of protein molecules immobilized on the tip, it is a challenge to obtain the effective number of protein molecules in direct contact with the surface. Thus, exploring an efficient way to quantify the AFM-measured interaction force is of great importance.

Raman is another advanced and largely used technique for the structural characterization of molecules by analyzing their vibrational transition spectra, which has penetrated to applications in electrochemistry, catalysis, biology, materials science, and others [27,28,29]. To further improve the sensitivity, surface-enhanced Raman scattering (SERS) has been developed and recognized as a sensitive analysis technique that features significantly amplified Raman signals, enabling even single-molecule detection [30]. SERS is also a powerful technique for obtaining the orientation and interaction of adsorbed protein on the active-substrates through measuring the vibrational spectra of protein molecules adsorbed on the surface [27,31]. Regulation of SERS active-substrates is often used to improve the sensitivity and selectivity for bio-detection [30], especially for the semiconductor-based materials used as SERS active-substrates [32,33,34,35] with a low SERS intensity, for instance, structural optimization, modification and composition. Meanwhile, adjusting the experimental conditions, including pH values [36,37], temperature [38,39], and ionic strength [40,41], is often used to regulate the interaction strength of the adsorbed proteins [42], leading to a significant increase in sensitivity [43]. Thus, developing different ways to improve the semiconductor-based SERS is crucial.

To gain a more quantitative description and understanding of the protein interactions with solid-surfaces at the molecular level, theoretical studies and simulations can be used for estimations [15,44,45,46,47]. MD simulations are an important tool for exploring the atomistic mechanisms involved in the adsorption process in complex biological systems [48]. In particular, it offers a method to analyze the synergic effects of surface nanomorphology and chemical compositions on the adsorption dynamics of proteins on nanostructured substrates [49]. Furthermore, the MD simulations can be applied to predict the adsorptive behavior and orientation of proteins, which provides a fundamental understanding at the molecular level.

In this review, the research on using these typical techniques to study the protein-TiO_2_-based porous material interactions at different scales was summarized and the scenarios and scales where the interfacial phenomena play a key role were focused on. The main techniques include high-performance liquid chromatography (HPLC) at macroscale, atomic force microscopy (AFM) at mesoscale, surface enhanced Raman scattering (SERS) at nanoscale, and molecular simulation (MD) at nanoscale. We intend to discuss different techniques for understanding the protein interaction with the widely-used TiO_2_-based materials.

## 2. Materials and Methods

### 2.1. HPLC-Based Techniques on Biomolecule-TiO_2_ Interactions

The separation and purification of proteins are important for DNA sequencing, disease surveillance, and biopharmaceutical analysis [50,51,52,53,54], which mainly relies on the different interaction strengths of each protein with solid surfaces. An improved understanding of the protein–surface interaction is critical for predicting and controlling chromatographic separations [55,56,57]. The columns of HPLC are usually packed with the materials of pellicular or porous particles. The pellicular particles are made of polymer or glass beads surrounded by a thin, uniform layer of silica, alumina, polystyrene-divinyl-benzene synthetic or other types of ion-exchange resins. Nawrocki et al. had published a comprehensive review as a general guide to metal oxide affinity chromatography, including a description of pH stability, particle preparation and surface properties, as well as chromatographic chemistry [58,59]. Due to its amphoteric nature, hydrolytic stability at extreme pH, higher isoelectric point (pI) value than silica, TiO_2_ has been used as an alternative material for column packing in HPLC [60]. Engholm-Keller et al. summarized the development of TiO_2_-based chromatographic strategies for separating different biomolecules from introducing small molecules over 20 years ago to proteomics applications in 2011 [61].

In 1990s, Kawahara et al. used TiO_2_ as column packing materials in HPLC to separate biogenic substances for the first time, revealing a high resistance of TiO_2_ to both alkaline and acidic eluents [62]. These porous TiO_2_ microspheres did further exhibit an excellent stability under high pressure and a variety of pH conditions, making them very suitable for HPLC applications. Chemie et al. compared the TiO_2_ sorbent for HPLC with silica, alumina, and zirconia sorbent concerning its physical and chemical properties, finding that the TiO_2_ sorbent enabled the separation of non-basic isomeric substance mixtures due to the Ti-OH groups on its surface [63]. Mazanek et al. chose TiO_2_ as a chemo-affinity solid phase in HPLC for the selective enrichment of phosphopeptides [64]. Wijeratne et al. used the TiO_2_ nanotubes grown radially on titanium wires and the commercial beads to separate phosphopeptides produced from complex tissue extracts of mouse liver [65]. The results showed that the TiO_2_ nanotubes provide comparable efficacy for the enrichment of phosphopeptides and have the potential to be a low-cost and practical material in biological studies.

**Figure 1:**
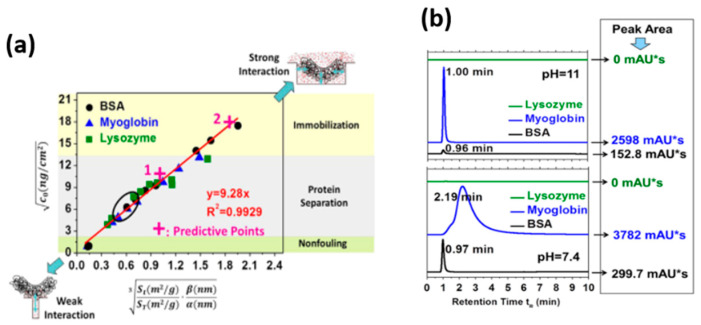
(**a**) A linearly predictive model between the geometry structure of mesoporous TiO_2_ and protein adsorptive behavior. (**b**) HPLC measurements of each protein samples after passing through the imprinted home-made TiO_2_ column. Reprinted from Ref. [66] with permission from Elsevier.

The structure of the material (surface area, pore size, etc.) is of great significance for the design of anti-fouling surfaces, chromatographic separation, and immobilized enzyme materials [6,8]. An et al. synthesized a new mesoporous TiO_2_ and prepared it as the home-made chromatographic column to study the retention behavior of different proteins [66]. Inspired by the phenomenon that different surface structures can significantly affect protein adsorptive behavior, they also demonstrated that the different pore size of TiO_2_ did alter the protein–surface interactions. They established a linearly predictive model between the geometry structure of TiO_2_ and protein adsorption (Figure 1a), and combined the chromatographic retention behavior with this model (Figure 1b). In this predictive model, the adsorption amount of proteins is translated into 1-dimensional interaction by squared root processing. Meanwhile, the geometrical parameters are combined due to the possibility of protein molecules contacting the TiO_2_ surface by cubic root processing. The HPLC findings further verified and demonstrated the accuracy of the predictive adsorption model. The results showed that the affinity for the different proteins onto TiO_2_ surfaces follows the order: lysozyme > BSA > myoglobin.

**Figure 2:**
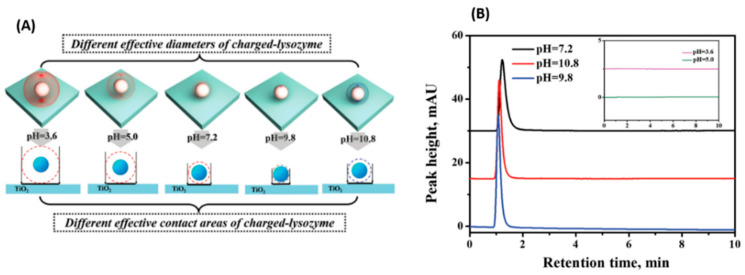
(**A**) Schematic diagram of the effective diameter of charged lysozyme, and the effective contact area of charged lysozyme with TiO_2_ under different pH conditions. (**B**) HPLC retention behavior of lysozyme under different pH conditions passing through the imprinted home-made TiO_2_ column. Reprinted from Ref. [42] with permission from Wiley.

Dong et al. examined the interaction strength of protein with mesoporous TiO_2_ at the macroscale under different pH conditions with the HPLC measurements. They also investigated the effect of pH conditions on the molecular force of the protein molecule with TiO_2_ to guide the HPLC measurements [42]. They found that the molecular force was related to the effective contact area of the charged protein molecule (Figure 2A), which further can be used directly to design the HPLC measurements by tuning the retention behavior of the protein under different pH conditions (Figure 2B), indicating that the retention behavior at the macroscale is related to the molecular force at the nanoscale and the larger the molecular force, the longer the retention time. Furthermore, they also studied the contributions of ionic strength on the protein−surface interaction, as well as the effects on the retention behaviors of the proteins tested by HPLC [67]. They found that the interaction between protein and TiO_2_ became weaker when the ionic strength is high, and the retention behavior of the proteins by HPLC can be effectively detected under high ionic strength, corresponding to the shorter retention time and larger peak area.

More and more TiO_2_-based hybrid materials have been widely used as chromatographic packing materials. Kupcik et al. reported using the amorphous TiO_2_ nanotubes (TiO_2_NTs) and the corresponding hybrid materials decorated with Fe_3_O_4_ nanoparticles (TiO_2_NTs@Fe_3_O_4_NPs) to achieve highly selective phosphopeptide enrichment [68]. Compared to those well-established TiO_2_ microsphere materials, the enrichment efficiency and selectivity of TiO_2_NTs and TiO_2_NTs@ Fe_3_O_4_NPs for phosphopeptides were increased to 28.7% and 25.3%, respectively. It indicated that both TiO_2_NTs and TiO_2_NTs@ Fe_3_O_4_NPs provided good physicochemical properties which are favorable for highly selective phosphopeptide enrichment. Shen et al. used the titania-coated silica core−shell hybrid composites (TiO_2_/SiO_2_) as adsorbent, and combined with a liquid chromatography−tandem mass spectrometry for extraction and quantification of phospholipids from shrimp waste [69]. Recently, Dong et al. used the choline-based amino acid ionic liquid (IL) as the trace additives and loaded on mesoporous TiO_2_ surface physically for the separation of two highly similar proteins (size, molecular weight, pI), lysozyme and cytochrome c [70]. The hydration properties of the ionic liquids loaded on the TiO_2_ surface increased as the pH increased from 5.0 to 9.8, further weakening the protein interaction strength with the TiO_2_ substrates, especially for lysozyme. They also studied the retention behavior of the mixed proteins passing through the TiO_2_ column using HPLC and found that the introduction of ionic liquids (ILs) can separate the two highly similar proteins effectively.

Thus, TiO_2_-based materials have been introduced as a popular material for affinity chromatography of biomolecules, where the behavior of biomolecules interacting with TiO_2_ materials can be adjusted effectively, and the scope of applications of TiO_2_-based chromatography in separation of various biomolecules has continuously expanded and increased.

### 2.2. AFM-Based Techniques on Biomolecule-TiO_2_ Interactions

Different to the techniques that determine the interactions at the macroscale, atomic force microscopy (AFM) has been used as an effective and popular tool to reveal the mechanisms and investigate the interactions at the molecular level. The interaction force is exploited to keep the distance between the tip and the sample as a constant [71]. The most important feature of AFM is its ability to image samples in liquids, which is important for biological studies. AFM can enable the imaging of biological interfaces from cellular to molecular scales, indicating that it can directly visualize adsorbed proteins on different surfaces. In addition to imaging the surfaces, AFM can be used to obtain the interaction force between the tip and the sample by measuring the tip perpendicular to the surface while obtaining the force-distance curves [72].

**Figure 3:**
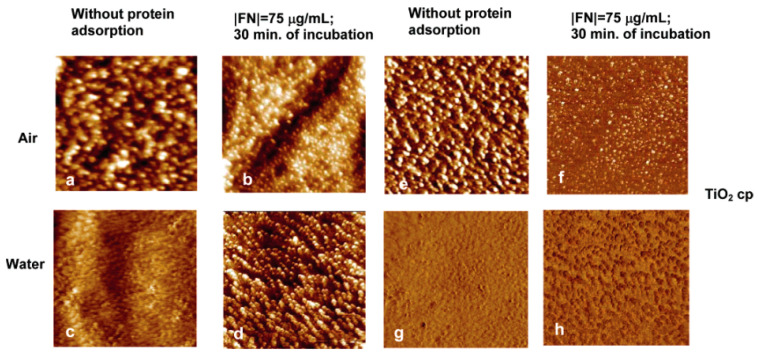
AFM height (**a**–**d**) and corresponding phase (**e**–**h**) images of TiO_2_ recorded in air and water before and after protein adsorption. Reprinted from Ref. [74] with permission from American Chemistry Society.

In 2000, Cacciafesta et al. studied the fibrinogen adsorption on the ultraflat TiO_2_ surfaces with AFM at molecular resolution [73]. The results showed that the ultraflat TiO_2_ surface allowed visualization at the molecular resolution of both individual fibrinogen molecules and aggregates, while inferring a stronger interaction between the protein and the TiO_2_ surface. Sousa et al. used AFM to perform the roughness and imaging analyses before and after protein adsorption. As shown in Figure 3, the roughness results revealed that the TiO_2_ surfaces exhibited a lower roughness in air than in water before protein adsorption, which is probably due to the effect of the previously reported formation of the gel-like TiO_2_ with a hydrated layer, rather than dehydrated oxides. While after protein adsorption, the height and phase images differed in morphology and contrast from the images on the surface without protein adsorption, and the roughness values were similar in air and in water [74].

Li et al. prepared the laccase-immobilized bacterial cellulose/TiO_2_ functionalized hybrid membrane, and the installation of both TiO_2_ and the functionalized hybrid membrane were confirmed by AFM [75]. The results showed that the surface was completely covered with laccase after the immobilization of laccase on the oxidized bacterial cellulose/TiO_2_. Since TiO_2_ is hydrophilic and has a high surface area, laccase can be easily loaded onto its surface. Meanwhile, the immobilized laccase showed better pH, temperature stability and reusability.

A newly developed AFM-based single molecule force spectroscopy (SMFS) has been established as a reliable standard method to study single-molecule interactions [76,77,78,79,80,81]. Hoffmann and Dougan published a tutorial review about the SMF, and introduced two main operation modes for the force spectroscopy: force-extension and force-clamp [80]. They described the use of polyproteins to obtain clear mechanical fingerprints to monitor the protein response to applied mechanical forces [80]. Ganbaatar et al. performed the surface force analysis on TiO_2_ with AFM by mounting a single amino acid residue on the AFM probe for the first time at the molecular level [82]. Force analysis on surfaces with three different crystal orientations revealed that the TiO_2_ (110) surface has the unique property of adsorbing glycine molecules, showing different characteristics compared to the TiO_2_ (001) and (100). Leader et al. used the single force spectroscopy to measure the adhesion forces between hydrophobic, aromatic and polar amino acids and TiO_2_ surfaces. Compared with hydrophobic and uncharged amino acids, the aromatic and positively charged amino acids dominate in the affinity to the TiO_2_ surface in this work [83].

AFM can be used to measure not only the conformational changes of proteins adsorbing on biomaterial surfaces, but also the interaction force between the proteins and the substrates by immobilizing the protein clusters onto the AFM tips according to the self-assembled monolayer (SAM) methods. Vergaro et al. investigated the interactions between human serum albumin (HSA) and different anatase TiO_2_ nanoparticles, and found that the adhesion forces depended on the degree of hydrophilicity of the TiO_2_ substrates. Surface roughness measurements showed that the molecules of HSA were arranged in a more globular manner on some of the nanocrystals. For nanocrystals with smaller primary particle size, lower protein affinity was found, which may correspond to their higher biocompatibility [84].

**Figure 4:**
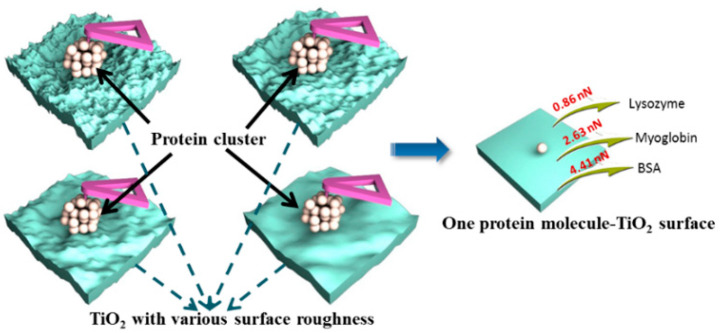
The molecular interaction of different proteins with TiO_2_ with different surface roughness decomposed from the total adhesion force. Reprinted from Ref. [85] with permission from the American Chemistry Society.

Although AFM can achieve a stretching of a single molecule or even measure a single bond [78,79], the results cannot reflect the overall effects of the solid surface at the nanoscale. This is because the solid surface, e.g., porous TiO_2_, with different surface structures such as pore size and roughness, was utilized to control the protein interaction by adjusting the effective contact area between the protein and the substrate. This indicated that the AFM-measured interaction force is a total force which is related to the surface structure-induced contact area. How to obtain the molecular interaction of one single molecule with the solid surface decomposed from the interaction between the clusters and solid surface measured by AFM is of great interest but faces a difficult challenge. Dong et al. have created a series of works to quantify the molecular force of proteins with TiO_2_ surfaces by combining the macroscopic experiment with microscopic measurements of AFM. In 2017, a new AFM-based approach was presented for the first time by Dong et al. to determine the molecular force between proteins and mesoporous TiO_2_ with different surface roughness [85]. They reported that the molecular force was independent of the surface topography of the materials, as shown in Figure 4. Furthermore, they also extended the study to examine the effect of pH conditions on the molecular force of protein molecule with TiO_2_ using AFM [42]. They found that the molecular force is proportional to the pH-induced different effective diameter of charged protein, which is mainly due to the different corresponding effective contact area of one charged-protein molecule on the TiO_2_ surface.

**Figure 5:**
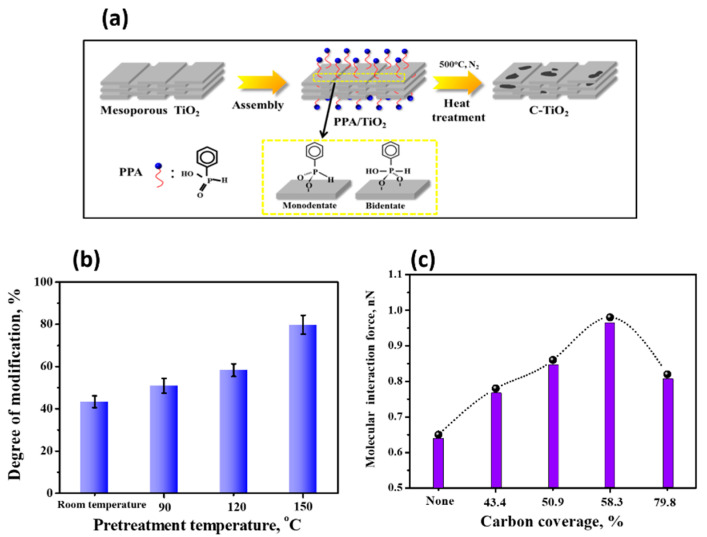
(**a**) Schematic diagram of the surface chemical modification of mesoporous TiO_2_ to obtain C-TiO_2_; (**b**) Different pretreatment temperatures corresponded to different carbon coverages of C-TiO_2_; (**c**) The relationship between the molecular force of protein molecule with C−TiO_2_ with different carbon coverages. Reprinted from Ref. [86] with permission from American Chemistry Society.

To improve the conductivity of TiO_2_, surface modification to form hybrid TiO_2_−carbon materials has been an efficient strategy. These hybrid TiO_2_−carbon materials simultaneously possess both the electron conductivity of carbon and the corrosion resistance of TiO_2_ materials. Dong et al. found that the molecular force of protein with TiO_2_ measured by AFM can be enhanced by the heterogeneous carbon modification of the TiO_2_ surface, as shown in Figure 5. The stronger interaction was due to the carbon modification being able to remove the -OH on the TiO_2_ surface, weaken the hydration ability, and further enhance the hydrophobicity of TiO_2_, leading to a stronger interaction with protein. The molecular force is independent of the surface roughness and structures but related to the chemistry of the C-TiO_2_, especially depending on the carbon coverages on the C−TiO_2_ surface, and a carbon coverage of 58.3% achieves the largest molecular interaction force [86].

Besides the modification of the surface chemistry of TiO_2_, using additives to form composite TiO_2_ substrate has been proposed as a desirable method to regulate the interactions between the adsorbed molecules and the related surfaces. Due to their unique physicochemical properties, including high ionic conductivity, tunable chemical structures and stability, ionic liquids (ILs) have rapidly been widely-used in the field of biological analysis and protein chemistry. Meanwhile, due to their highly heterogeneous micro-compositions, ILs can change the microenvironment to adjust the interactions [87,88]. Based on this, Dong et al. loaded ionic liquid (IL) on the TiO_2_ surface to investigate the protein interfacial interaction on the IL-TiO_2_ hybrid materials [89]. By quantifying the AFM-based adhesion force, the molecular interaction forces of cytochrome c with TiO_2_ and IL-TiO_2_ surfaces were determined. The molecular forces of protein with TiO_2_ for the systems, without IL, with IL in protein solution, and with ILs immobilized on the substrate, corresponded to the values of 1.65, 1.32, and 1.16 nN, respectively. It was shown that the molecular force was weakened after the addition of IL due to the hydration properties of the cation and anion of the IL.

The use of AFM has greatly advanced beyond high-resolution imaging to directly probe biomolecular interactions. Thus, AFM provides helpful insights into TiO_2_-based material design and protein-TiO_2_ interactions toward biological applications at the molecular level. Especially, the molecular force between the protein and the TiO_2_-based material quantified by AFM through decomposing from the total adhesion forces are of great significance. Extending this method to quantify the molecular interaction force of other molecules is worth exploring.

### 2.3. SERS-Based Techniques on Biomolecule-TiO_2_ Interactions

**Figure 6:**
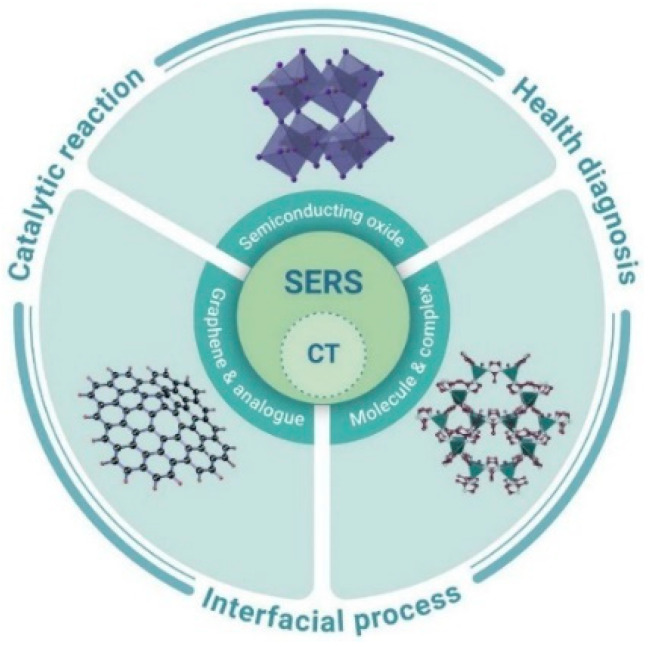
Schematic representing the charge-transfer (CT) induced SERS. The non-metal SERS substrates, including numerous types of semiconducting materials, such as semiconductor oxide, graphene and analogue, etc., were introduced with boosted SERS sensitivities. The promising applications include catalytic reaction, interfacial process, and health diagnosis, etc. Reprinted from Ref. [93] with permission from The Innovation.

SERS spectroscopy is an effective and advanced technique for detecting biomolecules, owing to its high sensitivity to molecular structures, and is a nondestructive analysis method. In terms of signal intensity, the active substrate plays an important role in affecting the SERS performance. Semiconductors, i.e., TiO_2_, ZnO, and Fe_3_O_4_, have been paid more and more attention to as SERS-active substrates, due to their superior properties, such as high stability, biocompatibility, and low cost [33,90,91,92]. A short review published by Cong et al. summarized the charge transfer transitions which are often the main contributors to the enhanced SERS activities in non-metal substrates, as shown in Figure 6 [93]. Based on the charge-transfer (CT)-induced SERS enhancement, a variety of semiconductor materials from inorganic to organic have been developed as novel SERS substrates [94].

**Figure 7:**
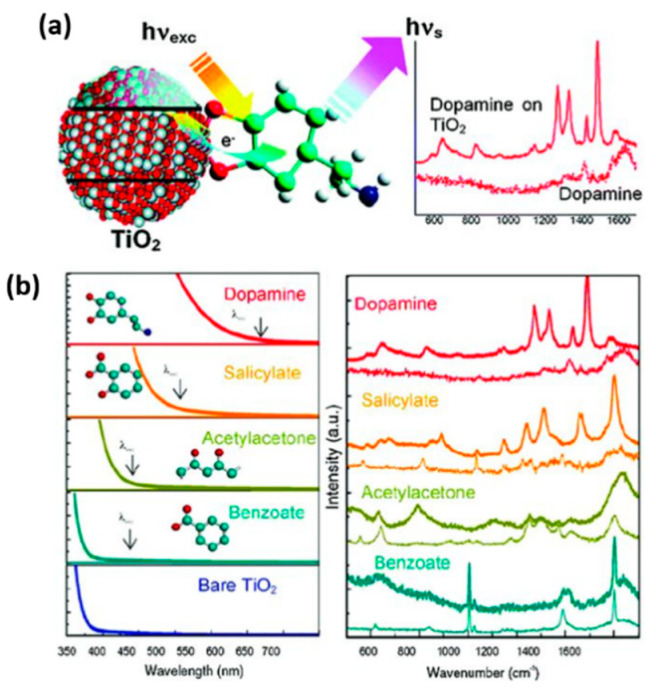
(**a**) Schematic illustration of SERS performance of dopamine adsorbed on TiO_2_ nanoparticles. (**b**) Absorption and SERS spectra of TiO_2_ nanoparticles modified with different ligands. Reprinted from Ref. [33] with permission from American Chemistry Society.

Typically, as the most studied semiconductor materials, TiO_2_-based materials have exhibited good SERS activity for detecting probe molecules [33,95,96,97]. Zhao et al. observed the SERS performance for the first time of the molecules adsorbed on the TiO_2_ nanoparticles in 2008, which is attributed to the charge-transfer mechanism of TiO_2_-to-molecule related to the surface state energy level of TiO_2_ [96]. They also systematically investigated the effects of different crystallinity of TiO_2_, pH conditions, and adsorption time on the SERS behavior and the interactions between adsorbed molecules and TiO_2_ [97,98]. Subsequently, Musumeci et al. observed strong enhancement of biologically active enediol molecules adsorbed on TiO_2_ nanoparticles with selected Raman active modes (Figure 7), and a molecule-to-TiO_2_ charge-transfer mechanism was also proposed to explain this enhancement [33].

However, since TiO_2_ is a low-conductivity semiconductor material, the detection sensitivity of TiO_2_ materials for protein detection is low [99]. Thus, many works focused on using TiO_2_ as a supporter of gold or silver to improve their SERS performances [100,101,102,103]. In addition to doping noble metals, the particle size, structure, surface defects, and optical band gap of substrates have been reported to have significant impacts on adjusting SERS performance [34,86,96,97,98,104,105]. Weidinger et al. prepared the TiO_2_ electrodes with different nanostructures as a platform for SERS and electrochemistry [104]. The SERS performance is ascribed to the enhanced local electric field derived from the synergistic effect of adjacent TiO_2_ nanorods. The results showed that the rougher surface could improve the SERS performance, and these nanostructured TiO_2_ electrodes exhibited an excellent electric field enhancement, which was due to the high anisotropy of the TiO_2_ nanostructure. Yang et al. observed that the SERS enhancements of probe molecules on TiO_2_ nanoparticles with different phase structures exhibit different degrees. Moreover, the mixed crystal structure TiO_2_ with a suitable ratio of rutile and anatase phase is favorable to SERS enhancement of molecules [97]. 

**Figure 8:**
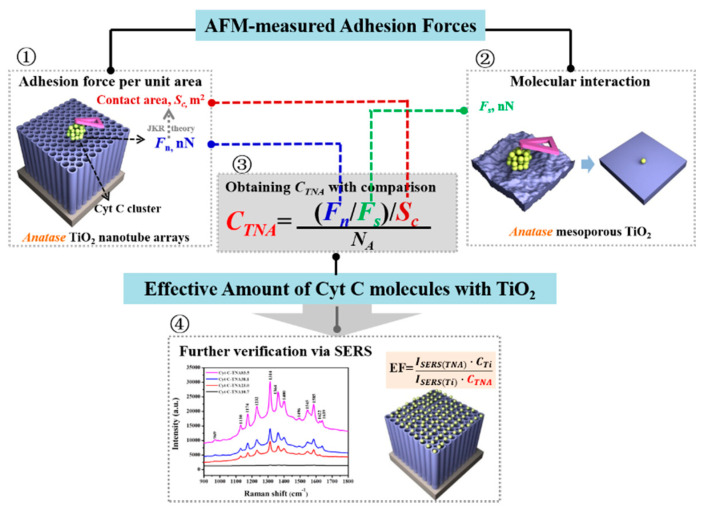
Scheme of the procedure for determining the effective amount of Cyt c molecules interacting with TiO_2_ nanotube arrays (③) by AFM-measured adhesion forces (①) and molecular force (②), and further verification by SERS and the calculated EF (④). Reprinted from Ref. [110] with permission from Elsevier.

As a unique structure, TiO_2_ nanotube arrays have attracted more and more attention and are used as a new kind of SERS-active substrate for protein detection, due to their intrinsically uniform structures, high stability, and electronic properties [100,101,102,103,106,107,108,109]. Öner et al. found that the TiO_2_ nanotube electrodes can preserve the structural integrity and redox behavior, and then they used these TiO_2_ nanotube electrodes with high biocompatibility and extraordinary spectroscopic properties to study the SERS performance of cytochrome b5 [108]. A strong SERS signal of the heme unit of cytochrome b5 was observed when the protein matrix was covalently immobilized to the TiO_2_ nanotube electrode. The higher SERS enhancement was attributed to the enhanced localized electric fields caused by the specific optical properties of the TiO_2_ nanotubular geometry. Meanwhile, the SERS enhancements generally depend on the features of the substrates, especially the surface roughness. Dong et al. found that the rougher surface of TiO_2_ substrates can adsorb more protein molecules and the SERS performance of Cyt c on TiO_2_ nanotubes can correlate quantitatively with the surface roughness of the substrates [110]. Furthermore, the enhancement factor (EF) is often regarded as a key standard in SERS, while accurately obtaining EF in SERS is a long-term problem, and the main challenge is to obtain the amount of proteins effectively excited by the laser [111,112]. In this article, the values of EFs were successfully calculated by combing the AFM-measured adhesion force and the quantified molecular force, further revealing that the effective amount of adsorbed proteins contributed directly to the protein interaction with the related electrode surfaces, as shown in Figure 8.

**Figure 9:**
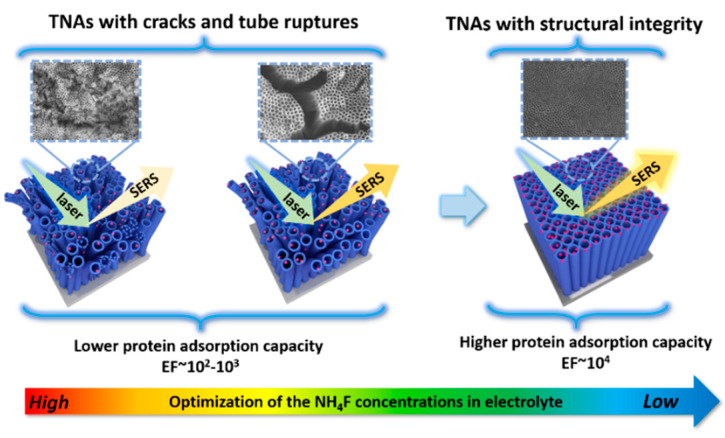
Schematic representation of SERS from Cyt c adsorbed on TiO_2_ nanotube arrays with different structural integrities through optimization of the topography. Reprinted from Ref. [113] with permission from American Chemistry Society.

Recently, Dong et al. proposed an effective and new method to optimize the topography and structure of TiO_2_ nanotube arrays through controlling the fluoride contents in the electrolyte. A significant enhancement of the SERS performance of Cyt c on these optimized TiO_2_ nanotube arrays was obtained, demonstrating the importance of the structural integrity of the nanotubular on achieving excellent SERS performance in the trace detection of proteins, as shown in Figure 9 [113]. This is because the fluoride contents in the electrolyte can affect the sizes of cracks and the tube ruptures of TiO_2_ nanotube arrays. It was found that the 0.2 wt % fluoride content can effectively provide the excellent and flat topography of TiO_2_ nanotube arrays. Meanwhile, the results also showed that the interaction force between protein and TiO_2_ nanotubes increased with decreasing the fluoride contents. They also found that the values of EF increased with the increase of the pore size of the TiO_2_ nanotube arrays, which is generally a key factor affecting the SERS performance [114,115,116,117]. It indicated that the structure and topography can affect not only the properties of TiO_2_ nanotubes, but also the interaction between the proteins and TiO_2_.

Besides using the pure TiO_2_ materials, Rajh et al. modified the semiconductors (e.g., TiO_2_, Fe_3_O_4_, et al.) with enediol ligands and found that the intensity of the SERS enhancement depends on the electron density of the ligands, the number of surface binding sites, and the dipole moment. The SERS performance was observed for the bioconjugated system, and the potential was further investigated for the system in developing Raman-based in vivo and vitro detection [118]. Zhao et al. prepared the TiO_2_/ZnO semiconductor heterojunction and found that the charge-transfer efficiency and SERS performance can be significantly improved, where an EF of 10^5^ can be achieved for a non-resonance molecule with the lowest detection concentration down to 10^−8^ M [119]. Das et al. prepared porous Ag–TiO_2_ film with nanocaged l structures as sensitive, recyclable, and low-cost SERS substrates to detect the various concentrations of blood urea for the first time. This new kind of porous Ag–TiO_2_ film can be used as a level-free biosensor for the analysis of biomolecules in biological and clinical applications [120]. Dong et al. modified the TiO_2_ with carbon to form the C-TiO_2_ hybrid materials and observed an enhanced SERS performance of cytochrome c on these C-TiO_2_ hybrid materials compared with the pure TiO_2_ [86]. This is because the modification can form the graphene types of carbon, further leading to an enhanced SERS performance [121]. Furthermore, Dong et al. also chose the ionic liquids as trace additives to synthesize the ionic liquid–TiO_2_ hybrid materials and used these hybrid materials as the active substrates to study the SERS performance of cytochrome c [87]. To the best of our knowledge, adding ionic liquids as additives into the SERS measurement to adjust the micro-environment has not been investigated before this work. The results showed that the determined EFs were 2.30 × 10^4^, 6.17 × 10^4^, and 1.19 × 10^5^, for the systems without ionic liquids, with ionic liquids in protein solutions and with ionic liquids immobilized on the substrate, respectively. These ionic liquid–TiO_2_ hybrid materials allowed the obtaining of an exceptionally low detection limit even down to 10^−9^ M. The mechanism is due to the dissociation and hydration of ionic liquids occurring in the SERS system; the hydration properties of the ionic liquids result in an improved electron transfer ability of ionic liquids and further lead to an excellent SERS performance in protein detection [122,123]. This work stimulates the development of the use of ionic liquids in SERS and related applications in bioanalysis and nanoscience.

SERS is a highly selective, sensitive, and versatile technique to achieve fast data acquisition, showing considerable promise for qualitative and quantitative analysis for life science. Studying SERS performance of adsorbed proteins on TiO_2_ meets the demands on trace detection in a variety of applications, also simulating the further development of TiO_2_-based materials on trace detections.

### 2.4. Molecular Simulations

**Figure 10:**
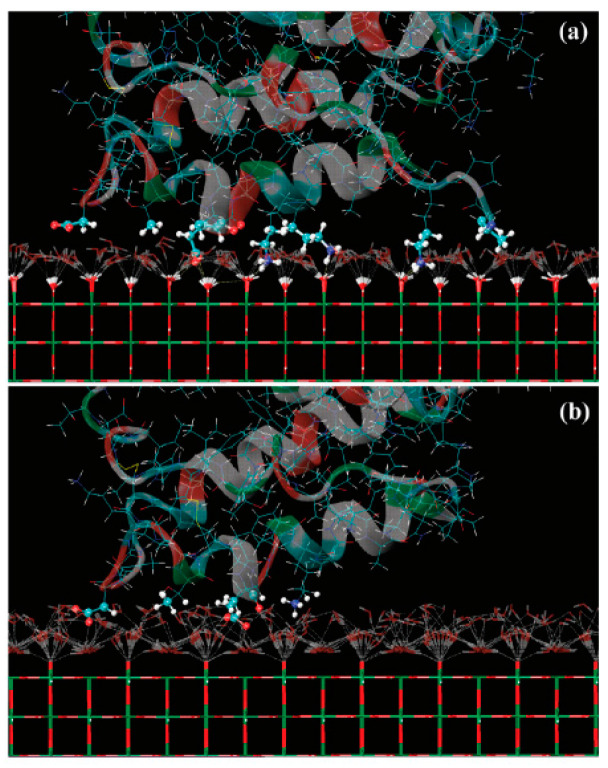
Snapshots of adsorption residues on (**a**) hydroxylated and (**b**) nonhydroxylated TiO_2_ surfaces. Only the interfacial water molecules with the hydrogen bond network are shown and are displayed as white dashed lines. Reprinted from Ref. [127] with permission from American Chemistry Society.

Experimental methods have been widely used to study the protein adsorptive behavior and interactions on a related surface, whereas molecular simulations are also well suited to provide insights into protein behavior on surfaces, i.e., orientation, conformation, and to providing molecular-level information [15,44,124]. A review of multiscale modeling and simulation methods for describing protein adsorption on surfaces with different properties was presented by Quan et al. [125]. The water molecules at the interfaces play a key role in protein adsorption. Guo et al. found that a sufficient amount of interfacial hydration near the surface of nanostructured TiO_2_ also promoted the attraction of fibronectin [126]. Kang et al. used molecular dynamics (MD) simulations to study the adsorption of human serum protein on non-hydroxylated and hydroxylated rutile TiO_2_ surfaces [127]. Their results indicated that the distribution of interfacial water molecules caused by surface modification plays an important role in protein adsorption. The TiO_2_ surface with the modification of hydroxyl groups was found to have a greater affinity to the protein, as shown in Figure 10. Zheng et al. studied the synergetic effects of hydroxylation state, surface nanostructure, and bioactive ions on the adsorption of collagen tripeptides to TiO_2_ surfaces using molecular dynamics (MD) simulations [128]. They found that a dense water layer on the non-hydroxylated surface prevented tripeptide adsorption, but the water exhibited a less ordered and more dispersed distribution on the hydroxylated surfaces, which was suitable for adsorbing tripeptides.

**Figure 11:**
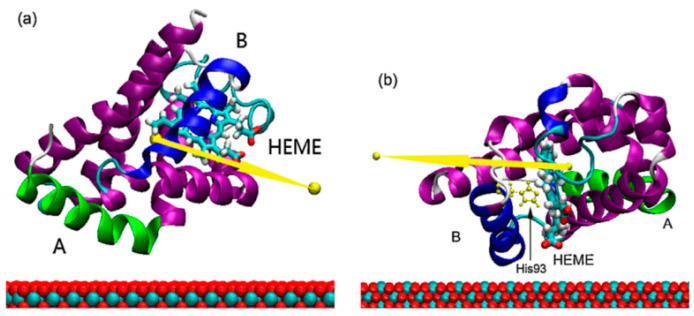
The adsorption configurations of myoglobin (**a**) on rutile TiO_2_ (110) and (**b**) on rutile TiO_2_ (001). The arrows represent the dipole direction of myoglobin. Reprinted from Ref. [131] with permission from Elsevier.

Wu et al. studied the conformational dynamics of the fibronectin adsorbed on the rutile TiO_2_ (110) surfaces by molecular dynamics (MD) simulations [129]. Their investigations showed that the binding strength and loss of protein secondary structure changed obviously, which depended on the surface topology of the substrate. Wu et al. also studied the binding of a negatively charged residue, aspartic acid, onto a negatively charged hydroxylated rutile TiO_2_ (110) surface in an aqueous solution, containing monovalent cations by molecular dynamics (MD) simulations [130]. Their results indicated that ionic radii and charges would significantly affect adsorption geometry, hydration, and distance of cations from the rutile surface, further leading to the regulation of the Asp/rutile binding mode. Yang et al. investigated the orientation and conformation of myoglobin adsorbed on rutile TiO_2_ (110) and (001) surfaces through the combination of the Monte Carlo and molecular dynamics (MD) methods [131]. Their simulation results showed that the adsorbed myoglobin conformations are not obviously affected by surfaces due to the strong hydrophilicity of both surfaces. However, the pathway of the electron transfer of myoglobin is closer to the rutile TiO_2_ (001) surface, which is favorable for achieving the faster electron transfer (Figure 11).

**Figure 12:**
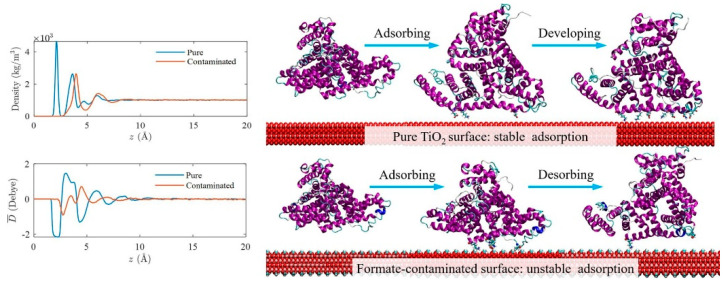
The density and dipole moment distributions of water molecules near the pure and formate contaminated surfaces; the scheme of the adsorption of BSA on pure TiO_2_ and formate contaminated TiO_2_ surface. Reprinted from Ref. [132] with permission from Elsevier.

Due to TiO_2_ being easily contaminated by carboxylic acid (i.e., formate) in ambient environments, Wu et al. studied the different adsorptive behaviors of BSA on pure and formate-contaminated rutile TiO_2_ (110) surface, respectively, for the first time [132]. They found that recontamination made further experimental studies difficult, despite numerous studies demonstrating that decontamination enhances albumin adsorption, which in turn improves hemocompatibility on TiO_2_ surfaces. Their MD simulation results showed that BSA could stably adsorb on the pure surface but the formate-contamination could decrease the TiO_2_ surface polarity and then the adsorption of BSA (Figure 12). Furthermore, they controlled the surface wettability by imposing the formate contamination on the rutile TiO_2_ (110) surface, and the adsorption properties of albumin and fibrinogen on the hydrophilic/hydrophobic TiO_2_ surface were systematically investigated by MD simulations [133]. Their results found that the albumin is favorable to adsorb on the hydrophilic surface due to the albumin having a higher proportion of charged residues. However, the fibrinogen is favorable to adsorb on the hydrophobic surface due to the hydrophobic surface being able to help the fibrinogen diffuse to the surface and adjust its orientation to achieve stable adsorption.

MD simulation is a great tool which can provide many microscopic mechanisms and phenomena that cannot be observed directly in experiments. Especially for TiO_2_-based materials, the simulation can provide detailed information on the effect of different crystal faces on the protein interaction, which behaves as a powerful tool to verify the interaction and clarify the mechanism at the molecular level.

## 3. Summary and Future Perspectives

In this review, different techniques for investigating the protein interaction with TiO_2_-based materials are presented, including the HPLC retention behavior, atomic AFM-based topographies and interaction forces, SERS-induced detection performance, and MD simulations of orientation, conformation, and molecular-level information. Quantifying the molecular force between protein and TiO_2_ under different realistic conditions (pH condition, surface structure and roughness, heterogeneity, etc.) is especially innovative, where the quantified molecular n forces can be used as a guideline at the nanoscale to design the experiments at the macroscale, such as HPLC-based protein separation, SERS-based protein bio-detection, and so forth. However, each technique is currently independent or qualitatively related due to the different scales studied. How to quantitatively correlate or combine these techniques at different scales is challenging. Researchers have also made many efforts to improve the combination of different techniques. For example, some in-situ techniques have been developed recently, such as Tip-Enhanced Raman Scattering (TERS) [134,135], which combines AFM and Raman with extremely high spatial resolution.

Moreover, the measured AFM-based interaction forces for the protein–solid surface systems can also be used to parameterize the force fields in molecular dynamics (MD) simulations. For such a complex interfacial system, MD simulations with all-atom force fields cannot fully grasp the interfacial properties, such as surface roughness and the adsorption of large amounts of proteins. The coarse-grained force field from AFM measurements could be an alternative for modeling the protein–solid interaction. Both the high precision AFM force measurement and the suitable model for a coarse-grained force field need to be advanced. Thus, it is expected that this review could be helpful for optimizing or combining these techniques for characterizing the interfacial behavior of protein on porous TiO_2_ materials in different practical applications.

In addition to the techniques summarized in this review, some other techniques can also be used to investigate the interactions of protein-TiO_2_ in the future. For example, Quartz crystal microbalance-dissipation (QCM-D), another advanced technique which enables real-time tracking of the adsorbed amount and viscoelastic properties of attached molecules at solid–liquid interfaces in biomolecular interaction processes [136,137,138]. Some optical techniques with the features of extremely high sensitivity have also been used to investigate the protein adsorptive behavior and trace detections, including ellipsometry, resonant waveguide gratings (RWG), surface plasmon resonance (SPR) [139], coupled plasmon-waveguide resonance spectroscopy (CPWR) [140], optical waveguide light-mode spectroscopy (OWLS) [141], and grating coupled interferometry (GCI) [142]. More and more advanced techniques are worth exploring to study the interactions between biomolecules and TiO_2_-based materials.

Furthermore, for studying the protein interaction with hybrid TiO_2_-based materials, introducing ionic liquids has been proved as an efficient method, i.e., ionic liquid-loaded TiO_2_ on separating proteins and the addition of ionic liquid on TiO_2_ on SERS detection. However, the analysis of the mechanisms still needs to be more in-depth. MD simulations could support understanding the mechanisms at the molecular level. Meanwhile, the classification of ionic liquids in the application of biological fields is also worth exploring. Furthermore, using different complementary methods to study more dynamic properties of protein with porous TiO_2_-based materials, including frictional behavior, lubrication, drug delivery, and so forth, should be discussed in a simple tutorial type of summary.

## Data Availability

Not applicable.
